# A potential therapeutic approach for ulcerative colitis: targeted regulation of macrophage polarization through phytochemicals

**DOI:** 10.3389/fimmu.2023.1155077

**Published:** 2023-05-01

**Authors:** Ke Wang, Tangyou Mao, Xinyu Lu, Muyuan Wang, Yifei Yun, Zeyu Jia, Lei Shi, Haoxi Jiang, Junxiang Li, Rui Shi

**Affiliations:** ^1^ Graduate School, Beijing University of Chinese Medicine, Beijing, China; ^2^ Dongfang Hospital, Beijing University of Chinese Medicine, Beijing, China

**Keywords:** macrophage, phenotype, polarization, phytochemicals, therapeutic effects, ulcerative colitis

## Abstract

Ulcerative colitis (UC), a type of inflammatory bowel disease characterized by recurring and incurable symptoms, causes immense suffering and economic burden for patients due to the limited treatment options available. Therefore, it is imperative to develop novel and promising strategies, as well as safe and effective drugs, for the clinical management of UC. Macrophages play a critical role as the initial line of defense in maintaining intestinal immune homeostasis, and their phenotypic transformation significantly influences the progression of UC. Scientific studies have demonstrated that directing macrophage polarization toward the M2 phenotype is an effective strategy for the prevention and treatment of UC. Phytochemicals derived from botanical sources have garnered the interest of the scientific community owing to their distinct bioactivity and nutritional value, which have been shown to confer beneficial protective effects against colonic inflammation. In this review, we explicated the influence of macrophage polarization on the development of UC and collated data on the significant potential of natural substances that can target the macrophage phenotype and elucidate the possible mechanism of action for its treatment. These findings may provide novel directions and references for the clinical management of UC.

## 1 Introduction

Ulcerative colitis (UC) is a chronic, idiopathic inflammatory disorder that affects the mucosa of the colon and rectum consecutively. It is listed by the World Health Organization as a modern refractory disease, with typical clinical symptoms including recurrent abdominal pain, diarrhea, and hematochezia. Over recent decades, the incidence rate of UC has surged globally, ranging from 0.5 to 31.5 per 100,000 individuals annually across various populations, according to relevant statistics. An escalating body of evidence has indicated that UC typically manifests in individuals aged 30 to 40 years, with no sex predominance (1–3). Despite significant advancements made in the past decades, the precise etiology of UC largely remains an enigma. Pathogenic factors, including genetic predisposition, environmental factors, intestinal dysbiosis, and immune dysregulation, are widely acknowledged as being involved in the development of UC (3, 4).

Lifelong treatment is almost imperative for patients diagnosed with UC due to the dearth of known preventive or fundamentally curative interventions. The current therapeutic goal is persistent remission, mucosal healing, and mitigation of the risk of colorectal neoplasia. As the major treatment option for UC, typical drugs used in the clinic mainly include aminosalicylates, corticosteroids, immunosuppressants, biological agents, and microecologics (5, 6), which have not achieved satisfactory results on account of the various adverse effects, drug tolerance, and the high rate of recrudescence, among others (7, 8). Hence, there is an urgent need to explore and develop novel safe and effective medications for UC.

Macrophages, as the sentinels of intestinal immune homeostasis, can manifest diverse functional phenotypes in response to various environmental cues and stimuli, thereby either promoting or resolving intestinal inflammation, which play an essential role in the development of UC. Based on the *in vitro* model of monocyte-derived macrophages, the macrophage population can be divided into the classically activated M1 macrophages with pro-inflammatory activity and the alternatively activated M2 macrophages with anti-inflammatory characteristics. In intestinal homeostasis, resident macrophages usually present an M2 phenotype with low reactivity to Toll-like receptor (TLR) ligands to maintain tolerance to the different antigens from food and symbiotic microflora (9). On the contrary, in the colon of patients with UC, M1 macrophages play a dominant role with the excessive accumulation of pro-inflammatory factors, leading to the damage of the intestinal epithelial barrier and the imbalance of immune homeostasis (10, 11). Furthermore, there is compelling evidence that macrophage polarization tends to be a severe imbalanced condition in the intestinal tissues of patients with UC who are non-responsive to conventional treatments (12, 13). Therefore, targeting macrophage polarization is often an attractive aspect for UC therapy.

## 2 Macrophage

Evidence indicates that intestinal resident cells are derived from embryonic precursors that undergo continuous *in situ* proliferation during the neonatal period and are subsequently replaced by macrophages originating from peripheral blood with advancing age (14, 15). Due to their constant exposure to gut pathogens and their high energy consumption, lamina propria (LP) macrophages have a short life span and necessitate continuous replenishment by bone marrow-derived peripheral blood monocytes, which subsequently differentiate into mature macrophages in the gut (16). The most abundant macrophage population in the body is distributed in the gastrointestinal mucosa, especially in the LP near the epithelium, while a small proportion exists in the smooth muscle layer of the intestinal wall (16). Maintaining gut immune homeostasis is a cooperative and an elaborately dynamic process that requires moderate tolerance for the beneficial commensal microorganisms colonizing the gastrointestinal tract and the innocuous antigens from food substances. At the same time, it is vital to respond promptly to invading pathogens for host protection (17). As a crucial component of innate immunity, intestinal macrophages are polarized to different phenotypes by environmental cues, with the heterogeneous functions of identifying pathogens, phagocytosing microorganisms and debris, remodeling impaired tissues, supporting regulatory T cells, and regulating inflammation, which are considered as the main factors contributing to and maintaining intestinal homeostasis (18–20). Throughout the construction of monocyte-derived macrophage models *in vitro*, the macrophage population can be classified into two categories with opposing functions: the classically activated macrophages (M1 macrophages) that represent pro-inflammatory conditions and the alternatively activated macrophages (M2 macrophages) that represent anti-inflammatory conditions (21–25). Once the balance of macrophage polarization is broken, dysfunction will occur, impairing the ability to maintain homeostasis and sense signals of tissue damage in the body, which may lead to inflammatory diseases (26). It is worth noting that, while this taxonomic approach substantially contributes to the comprehension of the metabolic programming of the different macrophage functions, it may not fully reflect the *in vivo* condition of macrophages, which are influenced by complex environmental cues and may display features of both phenotypes (9, 16). Research in this area has shown that when this occurs to certain cytokines or complexes such as transforming growth factor beta (TGF-β), glucocorticoids, or the immune complex, macrophages become a continuum of activation forms alongside the M1/M2 axis, with similar but different transcription and functions (9, 27). However, categorization into either M1 or M2 brings benefits in macrophage polarization to understand their heterogeneous functions and their transformation.

### 2.1 M1 macrophages

Generally, M1 macrophages could be activated by tumor necrosis factor alpha (TNF-α) and TLR ligands such as lipopolysaccharides (LPS) or interferon gamma (IFN-γ); overexpress CD64, CD86, and CD16/32; and secrete high levels of the pro-inflammatory cytokines TNF-α, IL-1α, IL-1β, IL-6, IL-12, and IL-23, among others. In terms of function, the M1 phenotype possesses antigen presentation, pathogen elimination, and antitumor abilities. These macrophages synthesize nitric oxide (NO), which can mediate protection against infection and reactive oxygen species (ROS)-induced tissue damage, as well as impair tissue regeneration and wound healing. Moreover, a large amount of inducible nitric oxide synthase (iNOS) is excreted by M1 macrophages, which is regarded as an antimicrobial cytokine (28, 29).

### 2.2 M2 macrophages

Representing anti-inflammatory activity, M2 macrophages are identified by distinct markers including IL-10, CD206, and CD163 (30) and are polarized by stimulating the Th2 cytokines IL-4 and IL-13 *via* the activation of STAT6 through IL-4 receptor alpha (IL-4Rα). In addition, IL-10 can also induce the M2 phenotype by activating STAT3 *via* the IL-10 receptor (IL-10R). Functionally, M2 macrophages have the potent capacity of phagocytosis, obliterate the debris of apoptotic cells, accelerate tissue repair and wound healing, and possess pro-angiogenic and pro-fibrotic properties. In addition, these macrophages produce higher levels of IL-10 and arginase 1 (Arg-1), an effector enzyme in urea metabolism that inhibits immune responses (29, 31).

## 3 The origination of intestinal macrophages

Intestinal resident macrophages are mainly supplemented by circulating monocytes recruited to the mucosa, which express lymphocyte antigen 6C-high (LY6C^hi^), CC-chemokine receptor 2-high (CCR2^hi^), and CX3C-chemokine receptor 1-low (CX3CR1^low^) in a mouse model. Subsequently, after entering the intestinal mucosa and encountering special intestinal signals such as TGF-β, macrophage colony-stimulating factor 1 (CSF-1 or M-CSF), and IL-10, as well as some environmental cues including short-chain fatty acids (SCFAs) produced by the gut microbiota (32), LY6C^hi^ monocytes begin to differentiate locally to mature resident macrophages. During this process, the monocyte population first occurs in major histocompatibility complex class II (MHCII) before increasing F4/80 with a decline in LY6C throughout a series of short-lived CX3CR1^int^ intermediates (33). Mature intestinal macrophages can be distinguished by F4/80^+^, CD11b^+^, CD11c^+^, and CD64^+^ with a high expression of CX3CR1 (34). Previous research has shown that, in intestinal homeostasis, the majority of resident macrophages exhibit weak reactivity for the stimulation of TLR, increased production of anti-inflammatory cytokines such as IL-10, and suppression of pro-inflammatory cytokines such as iNOS and IL-6 (32), which express CD163 and CD206 (35), characterized more like an M2 macrophage. These macrophages play a pivotal role in the regulation of gut homeostasis *via* the clearance of harmful bacteria and adventive substances, IL-10, and the excretion of prostaglandin E2 to stimulate epithelial stem cell renewal and survival to promote the integrity of the epithelial barrier (36).

Circulating monocyte differentiation in the context of inflammation occurrence has been changed and disrupted compared with the above description, which is switched to polarize into a pro-inflammatory condition, M1 macrophage. During this period, the terminal differentiation process of LY6C^hi^ monocytes into mature intestinal macrophages is disrupted, leading to the accumulation of LY6C^hi^ monocytes, LY6C^int^ population, MHCII-positive and CX3CR1^int^ immature macrophages. LY6C^int^CX3CR1^int^ cells retain their pro-inflammatory capacity through the secretion of inflammatory cytokines, including IL-12, IL-23, and IL-1β, thereby promoting type 1 T helper (Th1) and Th17 immune responses and aggravating tissue damage (32). There is evidence that the proportion and the number of CX3CR1^int^ macrophages obviously increase in mice after management with dextran sulfate sodium (DSS) (35).

Consequently, to some degree, Ly6C^lo^ monocytes could be regarded as the macrophages of the circulatory system. Monocytes gradually undergo differentiation into resident macrophages in intestinal homeostasis. When the colon is inflamed, resident macrophages are still replenished from monocytes in blood circulation. However, the phenotype changes from tolerant to sensitive to ambient and pro-inflammatory, with high TLR expression, and the balance of macrophage polarization is deflected to M1 macrophages. More interestingly, research has revealed that Ly6C^hi^ monocytes can also be transformed into Ly6C^lo^ monocytes and subsequently returned to the bone marrow to replenish the local macrophage population (9, 37). Nevertheless, the reasons underlying the dysregulation of macrophage polarization are not yet fully understood and may be related to the exceptional accumulation of monocytes and their response to local alterations or repolarization between M1 and M2 macrophages. Of course, it cannot be ruled out that both scenarios may act in concert (9, 35).

## 4 Macrophage polarization affects the development of UC

**Figure 1:**
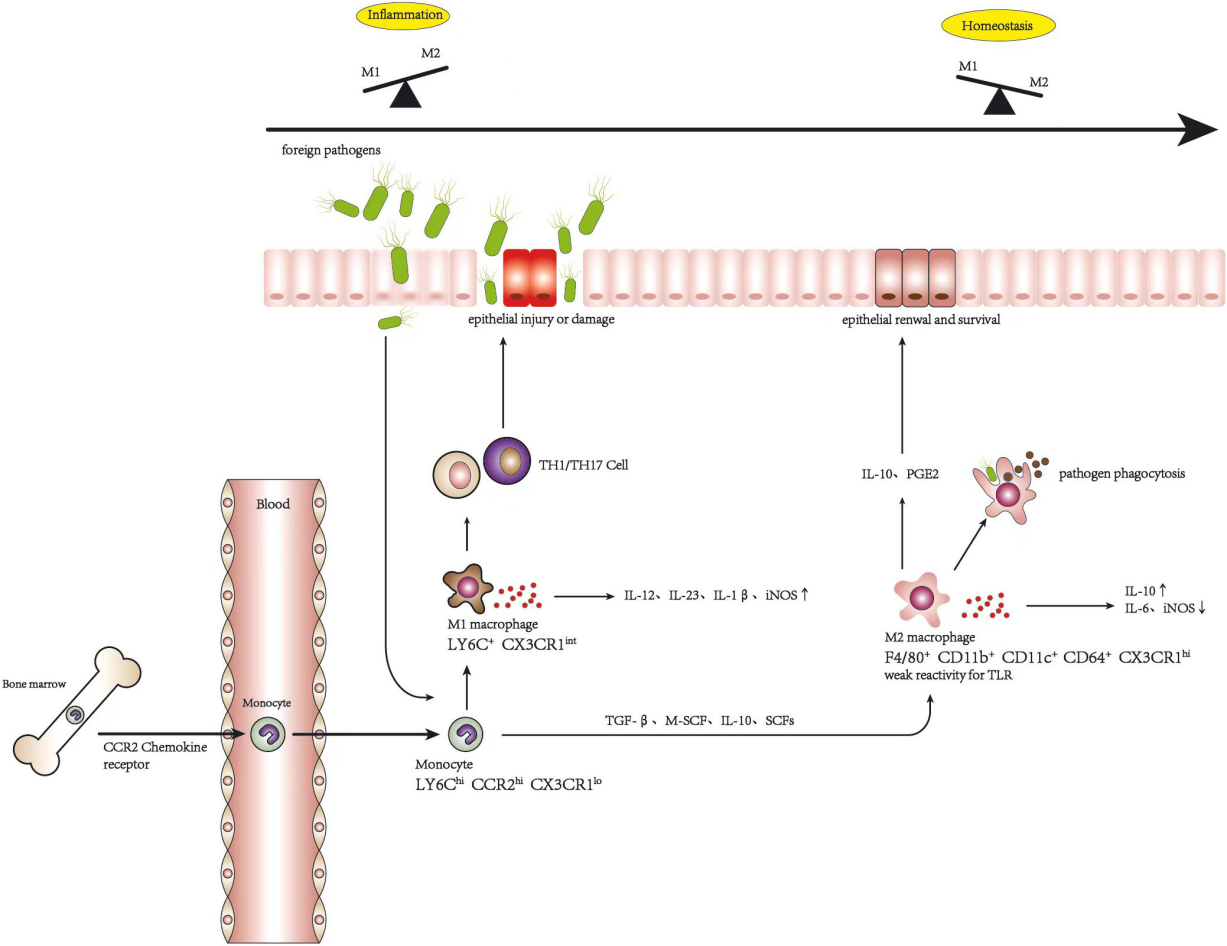
Alteration of macrophage polarization in inflammation and homeostasis.

The origination of macrophages posits that M1 and M2 macrophages are distinct subsets polarized from a common precursor, displaying diversity in phenotype and function, which can be polarized into various phenotypes in response to multiple environmental cues, consequently acquiring different abilities and transforming into each other under specific conditions (19, 35). CCR2^+^L6Cy^hi^ monocytes can replenish macrophages in all phenotypes during intestinal homeostasis and inflammation, which differentiate as a continuum of CX3CR1^int^ pool. UC is a chronic inflammatory disease driven initially by the disruption of the epithelial barrier, which is composed of a single layer of intestinal cells that connect with adjacent cells to form a continuous physical barrier, controlling the permeability of the luminal content (38). When foreign pathogens invade intestinal by crossing the injured epithelial layer, the balance of macrophages is deflected as M1 macrophages presenting a pro-inflammatory condition to engulf foreign materials and secrete pro-inflammatory cytokines such as TNF-α, IL-6, IL12, and IL-23, which promote immune responses mediated *via* Th1 and Th17 cells to protect the host from invasion (26). Under ideal conditions, the protective inflammatory response is self-limited and would completely resolve after the pathogens were eliminated, without causing tissue damage or impairment of wound healing due to excessive immune response. Generally, it is an active process controlled by the recruitment of M1 macrophages and the accumulation of M2 macrophages (32). However, the balance of macrophage polarization is destroyed gradually by the infiltration of more and more M1 macrophages, inflammatory cytokines are overexpressed, and a high level of iNOS is induced, which directly or indirectly affect intestinal epithelial cells, leading to their injury or necrosis, which elevates the occurrence and development of UC (9). The alteration of macrophage polarization in inflammation and homeostasis is shown in **Figure 1**. Furthermore, Lissner et al. revealed that M1 macrophages invaded intestinal deregulated tight junction proteins and induced epithelial cell apoptosis to disrupt the epithelial barrier directly (39). Correspondingly, there is evidence that the polarization of macrophages gives priority to the M1 phenotype in the intestinal mucosa of patients with inflammatory bowel disease (IBD) and in experimental colitis mice (11, 39, 40). It should be noted that, although colonic M1 macrophages predominate during colitis, M2-like resident macrophages are also present to combat inflammation and to facilitate wound healing, which helps resolve the inflammation (36). Indeed, promoting the phenotype of anti-inflammatory M2 macrophages has been considered a promising treatment for IBD. M2 macrophages play an important role in the alleviation of colitis.

## 5 Targeting macrophage polarization as an effective strategy for UC treatment

Given the significant impact of macrophage polarization on the development of UC, targeting the skewed axis represents a promising strategy for its prevention and treatment and has spurred extensive research. In recent years, studies have revealed that M1 macrophage enrichment in the colon biopsies of patients with IBD was positively correlated with disease severity (41). In addition, there is evidence that vedolizumab-induced reduction in the ratio of M1/M2 macrophages does contribute to the resolution of intestinal inflammation (42). It has been demonstrated that intraperitoneal or intravenous injection of exogenous bone marrow-derived M2 macrophages could effectively reduce the severity of colitis in mice caused by dinitrobenzene sulfonic acid (DNBS) (43, 44). Furthermore, it was evidenced that mice with deficient M2 macrophage polarization were more vulnerable to colitis induced by DSS (45). Caprioli et al. demonstrated that the downregulation of M1 macrophage pathway genes was connected to the mucosal healing of patients with IBD through treatment with infliximab, and M1 macrophages were significantly decreased, which was associated with the increased rate of macrophage apoptosis, representing a key mechanism for the therapeutic success of antitumor necrosis factor antibodies (46). Parallel results exist showing a prominent increase of the proportion of M2 macrophages in patients with IBD responding to infliximab therapy, but not in non-responders (30, 47). Eissa et al. have provided proof that the intestinal mucosa of patients diagnosed with UC is characterized by abundant infiltration of pro-inflammatory macrophages, producing a significant number of inflammatory mediators (e.g., TNF-α, IL-1β, and IL-6) through the activation of the nuclear factor kappa B (NF-κB) signaling pathway, which is negatively correlated with chromofungin (CHR), a short peptide with antimicrobial effects encoded from chromogranin A exon IV that is downregulated in UC. Moreover, exogenous CHR administration significantly mitigates colitis associated with a reduction of M1 macrophage markers (48). In addition, it has been revealed that intracolonic administration of CHR can increase M2 macrophage polarization, which decrease colonic collagen deposition and sustain the homeostasis of intestinal epithelial cells, thus protecting against colitis induced by DSS (49). Follistatin-like protein 1 (FSTL1), a pleiotropic cytokine that participates in a comprehensive spectrum of physiological and pathogenic processes, exhibits a highly expressional acitivity in human and mouse UC. It facilitates pro-inflammatory M1 phenotype macrophages and inhibits the M2 anti-inflammatory phenotype, leading to the excessive production of various inflammatory cytokines *in vitro* and *in vivo*. Li et al. found that the inhibition of FSTL1 could lead to UC remission, and the phenomenon disappears with the depletion of macrophages (50). Park et al. showed that adipose tissue-derived mesenchymal stem cells (ASCs) reduced the large amount of macrophages and the M1 macrophage population to mitigate UC in a model of DSS-induced mice. In the cell culture experiment, it was indicated that ASCs take effect by promoting the phenotype transition from M1 to M2, leading to anti-inflammatory cytokine proliferation (51). Cao et al. also reported that extracellular vesicles (EVs) secreted by bone marrow mesenchymal stem cells (BMSCs) could reinforce M2 macrophage polarization, supported by increased level of CD163 as the M2 marker, to effectively lessen the severity of UC. This result appeared to be associated with the JAK1/STAT1/STAT6 signaling pathway (52). These discoveries presume that the polarization of macrophages may be connected to mucosal healing in patients with IBD and could be an effective therapy for this disease.

## 6 Multiple phytochemicals show therapeutic prospects in UC treatment by targeting macrophage polarization

The first-line therapy currently used in the clinic for mild to moderate UC is mainly 5-aminosalicylic acid (5-ASA) drugs, which can be administered as suppositories, enemas, or oral preparations (53). Patients who do not respond to or do not achieve remission with 5-ASA drugs can be treated with corticosteroids (54), but glucocorticoids should not be used to maintain remission because of their lack of long-term efficacy and the risk of side effects (55). Thiopurines or biologic drugs, or both, should be used in patients with moderate to severe colitis, but long-term use must be carefully monitored for associated adverse effects, such as lymphoproliferative disorders (56, 57). Surgical treatment is usually indicated for uncontrollable massive bleeding, perforation, or endoscopically unresectable UC-associated adverse lesions (58). Phytochemicals, which are extracted from nature and with widely available sources, have been confirmed to possess abundant biological activities, as well as relatively low toxicity and high efficacy, which are particularly prominent in antitumor applications (59–61). The utilization of natural products in the management and prevention of various ailments can be traced back to ancient times, owing to their remarkable and indisputable effectiveness. The wealth of active compounds and diverse agent functions in natural products has always been an appealing prospect for researchers to explore and investigate novel phytochemical entity drugs with fewer adverse effects, thereby leading to more effective clinical application. In recent decades, a wealth of data has emerged indicating that numerous active ingredients sourced from plants and natural products hold immense potential in the management of IBD. This article aimed to present recent research focused on the treatment of UC using natural product-derived drugs that have been experimentally confirmed to be beneficial *in vitro* or *in vivo* based on online search protocols including PubMed, Web of Science, and Elsevier SD. The following key search terms were used: ‘ulcerative colitis,’ ‘Inflammatory bowel disease,’ ‘colitis,’ ‘Intestinal inflammation,’ ‘macrophage,’ ‘polarization,’ and ‘natural products,’ ‘compound,’ ‘phytochemical.’ The phytochemicals targeting macrophage polarization in UC treatment are listed in **Table 1**.

**Table 1:** Phytochemicals targeting macrophage polarization in ulcerative colitis (UC) treatment.

Phytochemicals	*In vitro*/*in vivo* model	Effective dose/concentration	Related clinical symptoms of UC	Macrophage phenotype-related indicators	Related molecular mechanisms in the regulation of macrophage phenotype in UC	Reference
*Dictyophora indusiata* polysaccharide	DSS-induced C57BL/6 mice	25, 50, and 100 mg/kg	Body weight loss↓ Colon length shortened↓ Histopathological damage↓ DAI↓ MPO↓ Spleen *vs*. body mass ratio↓	F4/80^+^CD11b^+^ cells in the spleen↓ F4/80^+^TNF-α^+^ cells in the spleen↓ F4/80^+^CD206^+^ cells in the spleen↑ TNF-α, IL-1β, and IL-6 levels and mRNA expression in the colon↓ IL-10 evel and mRNA expression in the colon↑	NLRP3, Bax, and IRF5 protein in the colon↓ p-STAT3/STAT3 in the colon↓ p-IκBα/IκBα in the colon↓ Bcl-2 and IRF4 protein↑ CD86 in the colon↓	(52)
Didymin	DSS-induced C57BL/6 mice	2 and 4 mg/kg	DAI↓ MPO↓ Histological scores↓ Colon length shortened↓ Infiltration of neutrophils in the colon↓	F4/80^+^Nos2^+^ cells in the colon↓ F4/80^+^CD206^+^ cells in the colon↑ Colonic TNF, IL-1β, IL-6, and Nos2 mRNA expression↓ Arg-1, Chil3, Retnla, and IL-10 mRNA expression↑		(55)
	LPS- and IFN-γ-induced BMDMs	3, 10, and 30 μM		F4/80^+^Nos2^+^ cells ↓ F4/80^+^CD206^+^ cells ↑ TNF, IL-1β, IL-6, and Nos2 mRNA expression↓ Arg-1, Chil3, and Retnla mRNA expression↑	OCR level↓ Acetyl-CoA level↑ Hadhb mRNA expression↑ Hadhb mRNA reverse F4/80^+^Nos2^+^ cells decreased, F4/80^+^CD206^+^ cells increased Hadhb mRNA inhibited the reduction of TNF, IL-1β, IL-6, and Nos2 mRNA expression and promoted Arg-1, Chil3, and Retnla mRNA expression	
	M2: IL-4- and IL13-induced BMDMs	3, 10, and 30 μM		No alteration in Arg-1, Chil3, and Retnla mRNA expression↑ IL-10 mRNA expression↑		
Genistein	DSS-induced C57BL/6 mice	10 mg/kg	Body weight loss↓ Colon length↑ Inflammation scores↓ Glandular cell architecture recovery CD4% in the spleen, cLP↓ CD4% in MLNs↑ DCs in the spleen, MLNs, cLP↑ CD4^+^IL-10^+^ T cells↑ in the cLP	CD11b^+^CD11C^+^ cells in the spleen, MLNs, cLP↓ F4/80^+^CD206^+^ cells in the spleen, MLNs, cLP↑		(60)
	F4/80^+^CD206^+^ cells sorted from the spleen			Arg1 and IL-10 levels↑		
Loganin	DSS-induced BALB/c mice	50 and 100 mg/kg	Body weight loss↓ DAI↓ Colon length ↑ MPO↓ Histological alterations↓ Inflammation infiltration↓	F4/80^+^iNOS^+^ cells in the colon↓ IL-6, TNF-α, and IL-1β mRNA expression and protein in the colon↓ MCP-1, CXCL10, and COX-2 mRNA expression in colon↓	Sirt1 mRNA expression in the colon↑ NF-κB p65 acetylation in the colon↓	(62)
Dioscin	DSS-induced BALB/c mice	40, 80, and 160 mg/kg	Body weight loss↓ DAI↓ Colon length↑ Histological scores↓ MPO↓	F4/80^+^CD86^+^ cells in the colon↓ F4/80^+^CD206^+^ cells in the colon↑ TNF-α, IFN-γ, and IL-6 levels in the colon↓ IL-10 level in the colon↑ CD86 protein in the colon↓ CD206 protein in the colon↑		(63)
	LPS- and IFN-γ-induced RAW264.7	0.625, 1.25, and 2.5 µM		F4/80^+^CD86^+^ cells↓ Expression of iNOS, TNF-α, and IL-6↓ Secretion of NO, TNF-α, IL-6, and IL-1β↓	Glucose, lactic acid↓ Protein of Raptor, HIF-1α, CD86, HK-2, PKM2, LDHA↓	
	RAW264.7	0.625, 1.25, and 2.5 µM		F4/80^+^CD206^+^ cells↑ Secretion of IL-10↑ Expression of Arg-1, IL-10, and Ym1↑	Uptake of free fatty acids↑ Protein of CD206, ACSL1, CPT-1A, CPT-2, Rictor, PPAR-γ↑	
Lupeol	LPS- and IFN-γ-induced peripheral blood mononuclear CD14^+^ cells in the presence of GM-CSF	5 and 10 μM		CD86^+^ cells↓ CD206^+^ cells↑ TNF-α and IL-1β levels↓ IL-12 and IL-10 levels↑	IRF5 protein↓ p-p38 protein↓ SB203580 (specific inhibitor of p38 MAPK) reduced IRF5 expression	(64)
	IL-4-induced peripheral blood mononuclear CD14^+^ cells in the presence of M-CSF	5 and 10 μM		No significant change in CD206^+^ cells and the IL-10 and IL13 levels in the colon	No significant change in IRF5 and p-p38 protein SB203580 (specific inhibitor of p38 MAPK) affected little in IRF5 protein	
	DSS-induced C57BL/6 mice	50 mg/kg	Histological scores ↓ Colon length↑ Body weight loss↓ Survival rate↑	mRNA expression of IL-12, IL-6, IL-1β, TNF-α, iNOS, and CD86 in the colon↓ mRNA expression of IL-10, IGF-1, and Arg-1 in the colon↑		
Berberine	DSS-induced C57BL/6 mice	40 mg/kg	Colon length↑ DAI↓ Inflammatory cell infiltration↓	F4/80^+^CD11b^+^CD16/32^+^ cells in the colon↓ TNF-α, IL-1β, and IL-6 levels in serum and mRNA expression the colon↓ IL-10 level in serum and mRNA expression the colon↑	AKT1 protein and mRNA expression in the colon↑ AKT2 mRNA expression in the colon↓	(65)
	LPS-induced RAW264.7	10 and 20 nM		CD16/32+cells↓ TNF-α, IL-12, IL-6 level and mRNA expression↓	AKT1 protein and mRNA expression↑ AKT2 mRNA expression in the colon↓ p-p65/NF-kB protein↓ SOCS1 protein↑ siAKT1 reduced CD16/32^+^ cells and SOCS1 promotion and p-p65/NF-kB decline by berberine siSOCS1 inhibited the reduction of p-p65/NF-kB by berberine	
Ginsenoside Rg1	DSS-induced BALB/c mice	200 mg/kg	Body weight loss↓ Colon length↑ Colon weight↓ Inflammatory cell infiltration↓	CD11b^+^F4/80^+^iNOS^+^ cells in the colon↓ CD11b^+^F4/80^+^CD206^+^ cells and CD11b^+^F4/80^+^CD163^+^cells in the colon↑ Arg1 protein in the colon↑ MIF-1 and PIM-1 protein in the colon↓	Rock1, RhoA, and Nogo-B proteins in the colon↓	(37)
Baicalin	LPS-induced mouse peritoneal macrophages	50 μM		TNF-α and IL-23 mRNA expression↓ Arg-1 and Fizz-1 mRNA expression↑	IRF4 siRNA inhibited iNOS/CD206 decliine by baicalin	(66)
	DSS-induced C57BL/6 mice	100 mg/kg	DAI↓ Inflammatory cell infiltration↓	iNOS/CD206 in the colon↓ TNF-α and IL-23 mRNA expression in the colon↓ Arg-1 and Fizz-1 mRNA expression in the colon↑ IRF4 protein in the colon↓ IRF5 protein in the colon↑		
Toosendanin	DSS-induced C57BL/6 mice	0.5 and 1 mg/kg	Colon length↑ Body weight loss↓ DAI↓ Weight of spleen↓ MPO↓ Histological score↓	CD11b^+^CD11c^+^ cells in the colon↓ F4/80^+^CD206^+^ cells in the colon↑ TNF-α, IL-6, and IL-1β levels and mRNA expression in the colon↓	NLRP3 protein↓ Nrf2 and HO-1 protein↑	(67)
Artemisinin	DSS-induced C57BL/6 mice	20 and 80 mg/kg	Body weight loss↓ Colon length↑ MPO↓ Inflammatory cell infiltration↓ DAI↓ Spleen index↓	TNF-α, IL-1β, and IL-6 levels in the colon↓ iNOS protein↓ Arg-1 protein↑	ERK phosphorylation↓ MyD88 activation↓	(68)
	PBMCs of CD patients	10 and 100 μM		CD11b^+^CD206^+^ cells↑ TNF-α, IL-1β, and IL-6 levels and mRNA expression↓		
Tiliroside	DSS-induced C57BL/6 mice	12.5, 25, and 50 mg/kg	Body weight loss↓ Diarrhea, rectal bleeding↓ Colon length↑ MPO↓ Inflammatory cell infiltration↓	CD68^+^iNOS^+^ cells in the colon CD68^+^CD206^+^ cells in the colon TNF-α, IL-1β, and IL-6 mRNA expression in the colon↓ Arg-1, Chil3, and CD206 mRNA expression in the colon↑		(69)
	TNBS-induced C57BL/6 mice	25 and 50 mg/kg	Survival rate↑ Colon length↑ MPO↓			
	LPS- and IFN-γ-induced BMDMs	10, 20, and 40 μM		TNF-α, IL-1β, and iNOS mRNA expression↓ Arg-1, Chil3, and CD206 mRNA expression↑	HIF-1α protein and mRNA expression↓	
	IL-4-induced BMDMs	10, 20, and 40 μM		No significant impact on Arg-1, Chil3, and CD206 mRNA expression		
Platycodin D	DSS-induced C57BL/J mice	10 mg/kg	Body weight loss↓ Colon length↑ DAI↓ Histological scores↓	F4/80^+^iNOS^+^ cells in the colon↓ F4/80^+^CD206^+^ cells in the colon↑ TNF-α, IL-1β, and IL-6 levels and mRNA expression in the colon↓ IL-10 level and mRNA expression in the colon↑		(70)
	LPS induced RAW264.7	2.5 and 5 μM		F4/80^+^iNOS^+^ cells↓ F4/80^+^CD206^+^ cells↑ TNF-α, IL-1β, and IL-6 levels and mRNA expression↓ IL-10 level and mRNA expression↑	p-PI3K and p-Akt protein↑ p-p65 protein↓	
Sulforaphane	DSS-induced C57BL/6JNifdc	10, 20, and 40 mg/kg	Body weight loss↓ Colon length↑ DAI↓ Histological scores↓	F4/80^+^CD68^+^ cells in the colon↓ F4/80^+^CD206^+^ cells in the colon↑		(71)
Rhein	M1:LPS + IFN-γ induced BMDMs	10, 1, and 0.1 μM		IL-1β and iNOS mRNA expression↓ IL-10 and CD206 mRNA expression↑ TNF-α, IL-1β, and IL-6 levels↓	p-STAT3 protein↓	(72)
Rosmarinic acid	DSS-induced ICR mice	100 mg/kg	Body weight loss↓ Inflammatory cell infiltration↓ DAI↓	TNF-α, IL-1β, IL-12, NOS2, and CD16/32 mRNA expression in the colon↓ Arg, Mrc, Mgl1, and CD206 mRNA expression in the colon↑		(73)
	LPS-induced PBMs	10 μM		TNF-α, IL-1β, IL-12, NOS2, and CCL4 mRNA expression↓	HO-1 protein↑	
	IL-4-induced PBM	10 μM		Arg, Mrc, Mgl1, and Dectin-1 mRNA expression in the colon↑		

PBMCs, peripheral blood mononuclear cells; BMDMs, bone marrow-derived macrophages; PBMs, peripheral blood macrophages; LPS, lipopolysaccharides; DSS, dextran sulfate sodium; DAI, Disease Activity Index; MPO, myeloperoxidase; cLP, colon lamina propria; MLNs, mesenteric lymph nodes; DCs, dendritic cells; iNOS, inducible nitric oxide synthase. ‘↑’ means increase, ‘↓’ means decrease.

### Dictyophora indusiata polysaccharide (DIP)

Dictyophora indusiata polysaccharide (DIP) isolated from dictyophora indusiate one of the most popular edible mushrooms due to its daintiness and multi-nutrition, was reported to possess potent antioxidant and anti-inflammatory activities *in vitro* (74, 75). Recent evidence has shown that DIP could conspicuously alleviate the severity of colitis in DSS-induced mice, and this mitigation is associated with the restoration of gut microbiota function and gut epithelial integrity, improvement of oxidative stress, and regulation of macrophage polarization balance (62, 76, 77). Wang et al. found that treatment with DIP significantly reduced M1 macrophage polarization and promoted the M2 phenotype in the spleen of mice orally administered DSS, and the macrophages marked by CD86 in the colon were also inhibited, which were consistent with the deregulation of the expression of TNF-α, IL-6, and IL-1β and the high secretion of IL-10 after DIP administration. In addition, DIP downregulated the activation of the NF-κB, STAT3, and NLRP3 signaling pathways in the colon of mice treated with DSS, which may be associated with the mechanism of macrophage polarization balance (62). Significantly, the biological activity of polysaccharides is highly correlated to their conformation of space, which means that their efficacy would greatly weaken or even vanish once they are degraded into monosaccharides or oligosaccharides.

### Didymin

Didymin, a dietary glycoside widely distributed in citrus fruits such as mandarin, bergamot, orange, *Origanum*, and Vulgare Duanxueliu, has attracted attention due to its antioxidant capacity (63). Recently, Lv et al. have found that didymin can effectively reduce colitis in mice by targeting macrophage polarization to the M2 phenotype. Their experiment results showed that didymin decreased the proportion of M1 and increased M2 in the colon of mice induced by DSS, and mice injected with exogenous M1 macrophages after being administered with clodronate liposomes to deplete autologous macrophages exhibited more sensitivity to DSS; however, the severity of colitis was declined by didymin management before exogenous M1 macrophages injection. Interestingly, didymin resisted M1 macrophage polarization, but there was no alteration on M2 macrophages and on the expression of Arg-1, Chil3, and Retnla, indicating that the effect of didymin on ameliorating colitis is dependent on the transformation of M1 macrophages toward M2. A further study suggested that the macrophage phenotype modulation of didymin is presented through the improvement of fatty acid oxidation (FAO) by fortifying the expression of Hadhb (60).

### Genistein

Genistein, an isoflavonoid compound widely distributed in soy-based products (78), also called phytoestrogen owing to a pattern resembling estradiol, shows high anti-inflammatory, anticancer, antioxidant, and antidiabetic properties and has attracted interest in medical research (64, 79, 80). Recently, it has been revealed that genistein could conspicuously mitigate experimental colitis, which is associated with targeting macrophage polarization. The results of the experiment indicated that the administration of genistein resulted in the decline of M1 and the elevation of M2 macrophages in the spleen, mesenteric lymph nodes (MLNs), and colon lamina propria (cLP) of DSS-induced mice. Apart from this, the M2 macrophages sorted from colitis mice induced by genistein highly expressed Arg-1 and IL-10 compared with those managed using phosphate-buffered saline (PBS). However, the detailed mechanism of how genistein shifts M1 macrophages toward the M2 phenotype still remains unclear (81).

### Loganin

Loganin, a type of bioactive iridoid glycoside extracted from traditional Chinese medicine, commonly called *Cornus officinalis*, was established to have potent anti-depression, neuropathic protection, and anti-inflammation effects (82–84). Yuan et al. reported that loganin could prominently alleviate the pathologic alterations of DSS-induced colitis, increase the tight junction proteins to protect the intestinal epithelial barrier, and inhibit the expression of colonic pro-inflammatory cytokines such as IL-1β, IL-6, and TNF-α (85). Another study demonstrated the high expression of Sirt1, inhibition of the acetylation of NF-κB p65, and the suppression of loganin in the M1 macrophages of colitis mice, which was counteracted after using the Sirt1 inhibitor Ex527. These findings suggest that the therapeutic potential may have involved the inhibition of M1 macrophages regulated by the Sirt1/NF-κB pathway (65).

### Dioscin

Dioscin is a steroid saponin isolated from *Dioscorea nipponica* (86), which has been reported to be a potential therapeutic component for colitis. It showed high availability in suppressing glycolysis and promoting FAO to predispose macrophage polarization from M1 toward M2. Interestingly, the agonist of the mTORC1 signal could reverse the effects of dioscin on the downregulation of glycolysis and the counteraction of the HIF-1α protein expression, which is indispensable for the transcription of the inflammatory cytokines and metabolic genes associated with glycolysis, resulting in the abortion of M1 macrophage decline. In addition, after administration of the mammalian target of rapamycin complex 2 (mTORC2) inhibitor, the enhancement of dioscin on the peroxisome proliferator-activated receptor gamma (PPAR-γ) protein and FAO-related enzymes was prominently impaired, and the promotion of M2 macrophages was counteracted as well. These findings demonstrated that dioscin modulated the polarization and metabolism of macrophages by regulating the mTORC1/HIF-1α and mTORC2/PPAR-γ signaling pathways to mitigate the severity of colitis, which was further confirmed in experimental colitis mice (87). Similarly, Shi et al. showed that dioscin catalyzed the expression of miR-125a-5p to shift macrophages toward the M2 phenotype, thereby restoring the intestinal epithelial barrier function and facilitating experimental colitis (88).

### Lupeol

Lupeol is a triterpenoid compound with exclusive bioactivity found in numerous natural plants including *Albizia lebbeck* and *Alnus glutinosa* (89). It has been reported that lupeol exhibited protective effects against colitis in experimental animals, and this involved blocking the NF-κB signaling of intestinal epithelial cells and modulating macrophages leaning toward the M2 phenotype to relieve inflammatory responses (66, 90). Zhu et al. observed that *IRF5*, a key transcription factor associated with M1 macrophages, was remarkably reduced after lupeol incubation of M1 macrophages induced by LPS and IFN-γ with exposure to granulocyte-macrophage colony-stimulating factor (GM-CSF), but the same results were not detected in M2 macrophages, which were inferred to be bound with the modulation of a specific signaling pathway. This hypothesis was subsequently confirmed as studies showed that the p38 mitogen-activated protein kinase (MAPK) phosphorylation of M1 macrophages was reduced by lupeol, which was counteracted by the use of the p38 MAPK inhibitor (90). Therefore, lupeol possibly inhibits *IRF5* through a specific receptor and downstream signaling pathway, such as p38 MAPK, to switch M1 macrophages toward M2.

### Berberine

Berberine, a plant isoquinoline alkaloid largely found in the root of *Coptis chinensis* (91), has been proven to be beneficial in colitis treatment through various mechanisms, such as inhibiting the IFN-γ and JAK2/STAT3 signaling pathways to attenuate inflammatory responses (67, 92), regulating the intestinal mucosal immune homeostasis through the Wnt/β-catenin pathway (93), and reducing the activation of the MAPK and NF-κB signaling pathways to decrease pro-inflammatory cytokine production (68). Recently, Yunxin et al. have reported that berberine could correct macrophage polarization imbalance by inhibiting differentiation of the M1 phenotype to prevent colitis development directly by upregulating the AKT1 pathway and the protein expression of *SOCS1*, one of the target genes of AKT1, and decreasing the level of NF-κB phosphorylation. In addition, it was observed that knocking out the AKT1 gene reversed the effect of berberine on the modulation of SOCS1 and NF-κB phosphorylation protein expression. The downregulation of berberine on M1 macrophage polarization and related pro-inflammatory cytokines, such as IL-6 and TNF-α, was also neutralized on account of *AKT1* small interfering RNA (siRNA) transfection, suggesting that the inhibitory activity of berberine on M1 polarization is dependent on the AKT1/SOCS1/NF-κB signaling pathway (94).

### Ginsenoside Rg1

Ginsenoside Rg1 is a major active constituent of *Panax ginseng* and has been reported to be an anti-inflammatory treatment for various diseases (69). Recent evidence has shown that ginsenoside Rg1 could conspicuously ameliorate the severity of symptoms and reduce the inflammatory response by downregulating the expression of TNF-α, IL-33, IL-6, and CCL-2 in a DSS-induced colitis mouse model (40, 95). Ginsenoside Rg1 has been reported to be a good regulator of macrophage polarization, which increased the M2 phenotype and inhibited the M1 phenotype, similar to Y27632, a specific inhibitor of Rock1. Furthermore, it is worth noting that the increase in the expression of the Rock1, RhoA, and Nogo-B proteins in the colonic tissues of colitis mice was attenuated by ginsenoside Rg1 and Y27632, demonstrating that the trends of Nogo signaling in the regulation of the macrophage phenotype in colitis mice were largely consistent with ginsenoside Rg1. These results may imply that regulation by ginsenoside Rg1 of the phenotype of macrophages in colitis mice may be associated with the Nogo-B signaling pathway (40).

### Baicalin

Baicalin, one of the active ingredients of *Scutellaria baicalensis* Georgi, was proven to be therapeutic in IBD (70, 96). Zhu et al. investigated the anti-inflammatory effect of baicalin against LPS-induced mouse peritoneal macrophages and found that it could effectively inhibit the LPS-induced promotion of the inflammatory macrophage subset of M1, reducing the ratio of M1/M2. Consistently, they found that baicalin treatment obviously mitigated the severity of DSS-induced colitis in mice (97). The related mechanism may involve the regulation of the IRF4/IRF5 protein expression of baicalin, as the results showed that baicalin could directly facilitate the protein expression of *IRF4* and block that of *IRF5*, and the decline of the M1/M2 of baicalin was reversed after *IRF4* siRNA transfection (97).

### Toosendanin (TSN)

Toosendanin (TSN) is a triterpenoid distributed in the bark or fruits of a type of commonly used Chinese herbal medicine, known as *Melia toosendan* Sieb et Zucc (59). Fan et al. determined that TSN could alleviate the symptoms of DSS-induced mice by reducing the inflammatory responses and macrophage polarization, as the experiment results showed that TSN could downregulate the percentage of the M1 phenotype and the expression of pro-inflammatory cytokines such as TNF-α, IL-6, and IL-1β, but promoted M2 macrophages. A further study revealed that the activation of NLRP3 induced by DSS in the colonic macrophage of colitis mice was reversed by TSN, which influenced the composition of IL-1β. Interestingly, TSN acted as an activator of the NFE-related factor 2 (*Nrf2*) signaling pathway, a key transcription factor facilitating the antioxidant response *via* the synthesis of heme oxygenase-1 (HO-1), which modulated IL-10 production to affect the macrophage phenotype (71). The results showed that the decline of the colonic expression of *Nrf2* and HO-1 in mice induced by DSS was counteracted by TSN management (98). This evidence implied that TSN regulation of macrophage alteration attenuating DSS-induced colitis is associated with the NLRP3 and Nrf2/HO-1 pathways, but the specificity of the relationship needs further validation.

### Artemisinin

Artemisinin is the main active compound isolated from *Artemisia annua* L, which was initially popular for its strong antimalarial properties, but which has also been revealed in recent years to exert various activities such as antivirus, anti-parasite, tumor suppression, and inflammation prevention (72). Previous evidence showed the protective function of artemisinin against DSS-induced colitis in mice, which involved the induction of CYP3A expression through the activation of the pregnane X receptor (PXR) (99). A study by Huai et al. provided proof that the inflammatory colonic tissues of patients with Crohn’s disease (CD) presented significantly increased M2 macrophages marked by CD11b^+^CD206^+^ and reduction of the pro-inflammatory cytokine expression after the administration of artemisinin *in vitro*. In addition, it has been suggested that artemisinin could mitigate the symptoms of colitis *via* upregulating the macrophages of murine colitis tissues polarized to the M2 phenotype, which may be associated with inhibiting the MYD88 and ERK signaling pathways owing to evidence showing that artemisinin significantly suppressed MyD88 activation and ERK phosphorylation in the colon tissue of a DSS-induced mouse model (100). However, the specific mechanism of artemisinin on the MYD88 and ERK signaling pathways affecting the regulation of macrophage phenotype remains to be further studied.

### Tiliroside

Tiliroside, a natural flavonoid derived from several medicinal and dietary plants, such as linden, rosehip, and strawberry, was revealed to exhibit anti-inflammatory, antioxidant, anticarcinogenic, and hepatoprotective activities (73). Zhuang et al. reported that the protective function of tiliroside in UC was related to the blocking of M1 macrophage polarization, and this effect was mainly achieved by accelerating the proteasomal degradation of HIF-1α, consequently attenuating glycolysis. This was validated in the experiments showing that tiliroside could significantly decrease the extraction of 2-NBDG, a fluorescent deoxyglucose analog widely used in detecting cellular glucose uptake; the gene expression of glycolytic enzymes such as glucose transporter 1 (Glut1), enolase 1 (Eno1), and pyruvate kinase M (Pkm); and the production of lactate in bone marrow-derived macrophages (BMDMs) induced by LPS and IFN-γ. Another evidence exhibited tiliroside prominently downregulating the protein level of HIF-1α, but had no effect on the mRNA expression. In addition, tiliroside ceased to be effective after using clodronate liposomes, which can significantly deplete macrophages *in vivo*, suggesting that tiliroside inhibited colitis through a macrophage-dependent mechanism (101). In a word, the findings above showed the potential of tiliroside as a therapeutic strategy for UC through targeting the HIF-1a/glycolysis pathway to mediate M1 macrophage reduction.

### Platycodin D (PLD)

Platycodin D (PLD) is a triterpenoid saponin extracted from the root of the *Platycodon grandiflorum* plant (102). Guo et al. studied the anti-inflammatory effects of PLD on DSS-induced colitis in mice, as well as on LPS-induced RAW264.7, and found that PLD was effective in mitigating colitis through shifting macrophage polarization to deflect the M2 phenotype. Further examination showed that the property of PLD on the regulation of macrophage polarization involved the activation of the PI3K/Akt pathway and the inhibition of the NF-κB pathway, as data revealed the upregulation of p-PI3K and p-Akt proteins with a decline of the nuclear translocation of the p65 subunit after PLD administration in LPS-stimulated RAW264.7 cells, which was further confirmed to be or at least partly dependent on adenosine 5′-monophosphate-activated protein kinase (AMPK) due to the effect of PLD on PI3K/Akt and NF-κB pathway modulation being reduced after the knockdown of AMPK. It is noteworthy that a higher dose of PLD exerted a lower anti-inflammatory effect on the macrophages managed by LPS compared to a lower dose, which may be associated with the modest suppression of cell activity (103).

### Sulforaphane

Sulforaphane is a dietary isothiocyanate widely distributed in cruciferous vegetables such as broccoli, cabbage, and Brussels sprouts. It possesses great antioxidant and anti-inflammatory activities (104). Studies on DSS-induced colitis in mice found that management with sulforaphane could conspicuously improve the clinical symptoms of colitis and the damaged epithelial integrity (105). Sun et al. reported that sulforaphane elevated the IL-10 production of LPS- and IFN-γ-induced BMDMs and switched the macrophages from the M1 to the M2 phenotype with the activation of STAT3. Moreover, after the neutralization of IL-10, the effect of sulforaphane on the M2 phenotype priority was suppressed, as well as the level of STAT3 phosphorylation, implying the modulation of sulforaphane on macrophage polarization mediating the phenotype switch from M1 to M2 in murine colitis caused by DSS, and this effect was closely related to the activation of the IL-10/STAT3 signaling pathways (106).

### Rhein

Rhein is a natural flavonoid compound derived from rhubarb that is widely used as a traditional Chinese medicine to treat edema, constipation, and inflammation (107). It has been reported that rhein has potential in alleviating DSS-induced colitis through regulating macrophage polarization toward the M2 phenotype, i.e., toward the anti-inflammatory condition. Experimental data from RAW264.7 cells showed that the relative expression of M1 markers and pro-inflammation mediators were significantly inhibited after the administration of rhein, but the results for M2 were totally reversed. In addition, researchers found that rhein could prevent the activation of the Nox2 redox-mediated NLRP3 inflammasome and modulate the Nrf2-dependent redox balance to block the maturation and secretion of IL-1β in macrophages, one of the key pro-inflammatory cytokines (108).

### Rosmarinic acid (RA)

Rosmarinic acid (RA) is a natural compound extracted from plants of the Lamiaceae family, including rosemary, lemon balm, and mint (109). Some studies have investigated the protective effects of RA against colitis in mice induced by DSS and found that it is a potential anti-inflammatory candidate for UC treatment (110, 111). Mai et al. reported that RA could inhibit M1 macrophages with the promotion of M2 in both the colonic tissues of DSS-induced mice and in peripheral blood macrophages cultured *in vitro* and upregulate the protein level of HO-1. In addition, the inhibition of RA of the LPS-mediated NF-κB p65 translocation into the nucleus was shortened by interdicting HO-1; moreover, the administration of the NF-κB inhibitor BAY11-7082 had no significant effect on the modulation of macrophage differentiation by RA. These results indicated that RA dampened M1 macrophage polarization *via* promoting HO-1 to impede the NF-κB pathway in ameliorating experimental colitis (111).

## 7 Conclusion and future perspectives

As a modern refractory disease, UC has negatively impacted the quality of life of patients due to its recurrence and obstinacy, with intolerable symptoms such as frequent hematochezia and abdominal pain (58). A series of studies have demonstrated the great importance of the imbalance of macrophage polarization in the development of UC; therefore, targeting macrophage polarization tendency to the anti-inflammatory phenotype, i.e., of M2, is a potential therapeutic option for UC (21, 30, 36, 112). As the hotspot of new drug development, natural products exhibit abundant bioactivities and nutritional value. In this paper, we summarized more than a dozen investigated phytochemicals extracted from diverse plants, including didymin, genistein, loganin, etc., which could ameliorate experimental colitis by modulating macrophage polarization (60, 65, 81). Their chemical structures include flavonoid, polyphenol, alkaloid, and terpenoid derivatives, and the related modulatory mechanisms involved regulating Hadhb-mediated FAO, the Sirt1/NF-κB signaling pathway, and mTORC2/PPAR-γ signaling, among others (60, 65, 87). The aforementioned findings demonstrated that phytochemicals have promising prospects in mitigating the symptoms of UC by modulating macrophage polarization. However, investigations pertaining to their therapeutic efficacy in patients with UC are yet to be conducted, as all current research has been limited to experimental animal models. Furthermore, the underlying regulatory mechanisms and the potential toxicity of these phytochemicals, which act as regulators of macrophage polarization, require further elucidation. Consequently, further research should concentrate on the toxicity and safety of phytochemicals with effects on the regulation of macrophage polarization, as well as on particular mechanisms that are needed to promote natural regulators of macrophage polarization as UC therapy.

## Author contributions

RS, JL, and TM provided direction and guidance for this manuscript. KW wrote the whole manuscript. XL, MW, YY, LS, ZJ, and HJ were responsible for the collation of the paper. RS and TM made significant revisions to the manuscript. All authors contributed to the article and approved the submitted version.
